# Supplementary material for: Bta-miR-6531 Regulates Calcium Influx in Bovine Leydig Cells and Is Associated with Sperm Motility
Source: Genes (Basel). 2022 Oct 3;13(10):1788. doi: 10.3390/genes13101788 (PMC9601708; doi:10.3390/genes13101788)
Supplement: Supplementary file 1 [file genes-13-01788-s001.zip › genes-1855997-supplementary.pdf]

Tables

Table S1. the primers are used in this study

Primers	Sequence (5'-3')
gene-MIR6531 F	GCAGAGCGTGTCTTAGTTA
gene-MIR6531 R	CCGTCAGGTACTCCAACA
bta-miR-6531 (cDNA synthesis)	GTCGTATCCAGTGCAGGGTCCGAGGTATTCGCACTGGATACGACTCTCTC
bta-miR-6531 (qPCR)	GCGCGTAGACTTGGTGTACTTT
URP	AGTGCAGGGTCCGAGGTATT
U6 F	CTCGCTTCGGCAGCACA
U6 R (cDNA synthesis)	AACGCTTCACGAATTTGCGT
Bta-miR-6531 mimics	Sense UAGACUUGGUGUACUUUGAGAGA<br>Antisense UCUCAAAGUACACCAAGUCUAUU
Bta-miR-6531 inhibitor	UCUCUCAAAGUACACCAAGUCUA
NC mimics	Sense UUCUCCGAACGUGUCACGUTT<br>Antisense ACGUGACACGUUCGGAGAATT
NC inhibitor	CAGUACUUUUGUGUAGUACAA
miR6531-F-BamH I	cgGGATCCAGAGCGTGTCTTAGTTA
miR6531-R-EcoR I	cGAATTCCCGTCAGGTACTCCAACA
ATP2A2-seed-F	acgcGTCGACggcaggcatcagagtcatcat
ATP2A2-seed-R	attGCGGCCGCtgttgtatatggcccgtcct
ATP2A2-mut-F	CAgggggggAAAATTGTAGAATTTCTTCAGTCGTTTG
ATP2A2-mut-R	ACAATTTTcccccccTGGGAAGGTTCAACTCGAGCA
GAPDH-F	GATGGTGAAGGTCGGAGTGAAC
GAPDH-R	GTCATTGATGGCGACGATGT
ATP2A2-F	CAGGCATCAGAGTCATCAT
ATP2A2-R	GGTGGAGAAGTTGTCATCA
siATP2A2	Sense CAGCUAUGACUGGAGACGGUGUAAA<br>Antisense UUUACACCGUCUCCAGUCAUAGCUG
siRNA control	Sense CAGAGUAGGUCCAGAGUGGUUCAAA<br>Antisense UUUGAACCACUCUGGACCUACUCUG

Table S2 the sperms samples used in WB analysis

	Bulls' ID	Fresh motility (%)	Frozen motility (%)
Low motility	1	62.4	36.25
	2	62.4	28.33
	3	62.75	35.12
Mean±SEM		62.15±0.16	33.24±3.50
High motility	4	73.46	44
	5	73.63	49.44
	6	73.72	46.11
Mean±SEM		73.60±0.11	46.52±2.24

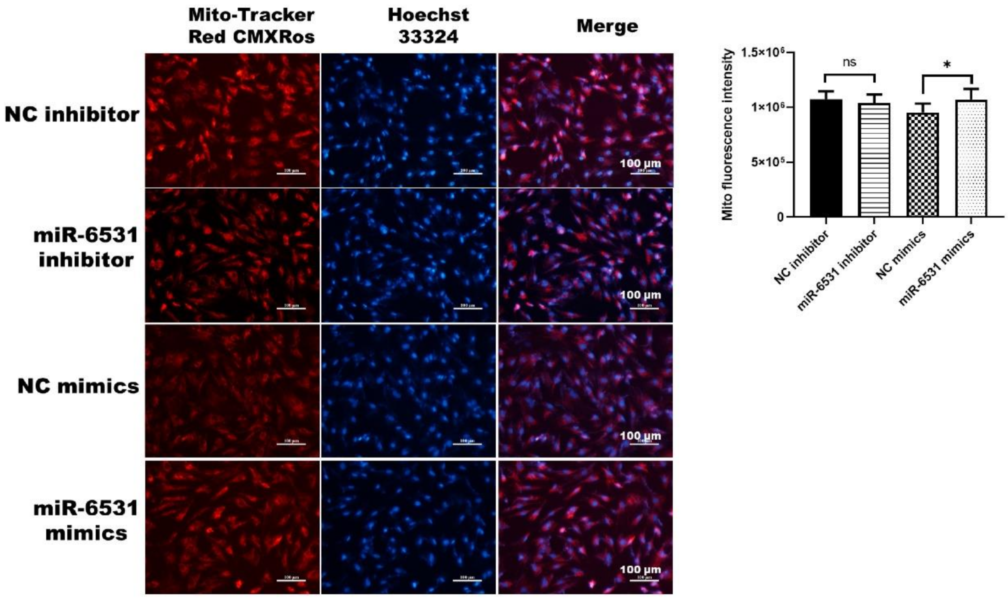

Figure S1. Measurement of MMP using fluorescent probe in Leydig cell.

Bovine Leydig cells were transfected with NC mimics or bta-miR-6531 mimics, and NC inhibitor or bta-miR-6531 inhibitors over 48 h, the cell MMP were measured. The mitochondria are marked by MitoTracker Red CMXRos, and nuclear is stained by Hoechst 33342.
